# Genetic alterations associated with multiple primary malignancies

**DOI:** 10.1002/cam4.3975

**Published:** 2021-05-31

**Authors:** Jenny Nyqvist, Anikó Kovács, Zakaria Einbeigi, Per Karlsson, Eva Forssell‐Aronsson, Khalil Helou, Toshima Z. Parris

**Affiliations:** ^1^ Department of Surgery Skaraborg Hospital Lidköping Sweden; ^2^ Institute of Biomedicine Sahlgrenska Academy University of Gothenburg Gothenburg Sweden; ^3^ Department of Clinical Pathology Sahlgrenska University Hospital Gothenburg Sweden; ^4^ Department of Medicine Southern Älvsborg Hospital Borås Sweden; ^5^ Department of Oncology Sahlgrenska University Hospital Gothenburg Sweden; ^6^ Department of Oncology Institute of Clinical Sciences Sahlgrenska Center for Cancer Research Sahlgrenska Academy University of Gothenburg Gothenburg Sweden; ^7^ Department of Medical Radiation Sciences Institute of Clinical Sciences Sahlgrenska Center for Cancer Research Sahlgrenska Academy University of Gothenburg Gothenburg Sweden; ^8^ Department of Medical Physics and Biomedical Engineering Sahlgrenska University Hospital Gothenburg Sweden

**Keywords:** breast cancer, double cancer, genome‐wide profiling, multiple primary malignancy

## Abstract

Breast cancer (BC) patients are frequently at risk of developing other malignancies following treatment. Although studies have been conducted to elucidate the etiology of multiple primary malignancies (MPM) after a BC diagnosis, few studies have investigated other previously diagnosed primary malignancies (OPPM) before BC. Here, genome‐wide profiling was used to identify potential driver DNA copy number alterations and somatic mutations that promote the development of MPMs. To compare the genomic profiles for two primary tumors (BC and OPPM) from the same patient, tumor pairs from 26 young women (≤50 years) diagnosed with one or more primary malignancies before breast cancer were analyzed. Malignant melanoma was the most frequent OPPM, followed by gynecologic‐ and hematologic malignancies. However, significantly more genetic alterations were detected in BC compared to the OPPM. BC also showed more genetic similarity as a group than the tumor pairs. Clonality testing showed that genetic alterations on chromosomes 1, 3, 16, and 19 were concordant in both tumors in 13 patients. *TP53* mutations were also found to be prevalent in BC, MM, and HM. Although all samples were classified as genetically unstable, chromothripsis‐like patterns were primarily observed in BC. Taken together, few recurrent genetic alterations were identified in both tumor pairs that can explain the development of MPMs in the same patient. However, larger studies are warranted to further investigate key driver mutations associated with MPMs.

## Graphical abstract

Genomic profiles for the tumor pairs for breast cancer patients with multiple primary malignancies.

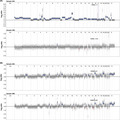

## 1 INTRODUCTION

Despite early detection and improved treatment regimens, cancer survivors have an increased risk of developing multiple primary malignancies.^1^, ^2^, ^3^, ^4^, ^5^, ^6^ According to Frödin and colleagues, 11% of patients registered in the Swedish Cancer Registry for a malignant tumor in 1988 were found to be diagnosed with more than one primary malignancy between 1958 and 1988.^7^ Aging is a key contributing factor to the increasing cancer incidence rates due to the accumulation of genetic and epigenetic changes during an individual's lifetime.^8^, ^9^, ^10^ Furthermore, cancer treatment may also contribute to the development of MPMs, as conventional radiation therapy, chemotherapy, and antihormonal therapies are associated with an elevated risk of developing angiosarcoma, hematopoietic malignancies, and gynecological malignancies, respectively.^1^, ^11^, ^12^, ^13^ However, the majority of studies on MPMs to date have focused on specific cancer types.^14^, ^15^, ^16^, ^17^

In 2018, over 2 million new breast cancer cases were reported worldwide.^18^ Nevertheless, little is still known about factors contributing to the development of MPMs diagnosed before BC. Previous studies have primarily focused on MPMs after a BC diagnosis, suggesting that the BC treatment itself may have contributed to the development of additional primary malignancies.^1^, ^11^, ^19^ In two recent studies, we demonstrated an increasing prevalence of gynecological tumors (endometrium and ovarian adenocarcinomas), malignant melanomas, and gastrointestinal malignancies diagnosed before BC in Swedish patients.^4^, ^5^ Therefore, these patients may be genetically predisposed to developing several primary malignancies as a result of cancer susceptibility genes and/or genes involved in maintaining genomic stability. Ghoussaini et al reported 72 inherited loci associated with BC susceptibility, of which 17 were associated with MPMs.^20^ Well‐known mutations and syndromes linked to BC and other MPMs include hereditary germline mutations in *BRCA1*/*2* and *PTEN* (Cowden's syndrome; both breast and thyroid cancer), and *TP53* (Li‐Fraumeni syndrome; breast cancer, sarcoma, brain cancer, and leukemia).^3^, ^21^, ^22^ Further, a specific founder mutation in *BRCA1* in Western Sweden has previously been defined by and may possibly contribute to MPM in the breast cancer population in Western Sweden.^23^

Guidelines for the clinical management of cancer patients frequently include testing for somatic mutations and histopathologic markers.^24^, ^25^, ^26^ However, we still need to have a better understanding of genetic alterations contributing to the development of OPPMs before a BC diagnosis. In the present study, we performed genome‐wide screening of the BC and OPPMs for 26 young women (≤50 years at the time of the BC diagnosis) to identify common DNA copy number alterations and somatic mutations in the tumor pairs. In addition, the genomic profiles were used to assess genomic instability, thereby identifying potential biomarkers for future screening programs.

## 2 MATERIALS AND METHODS

### 2.1 Patients and tumor samples

Of the 8,031 patients diagnosed with primary breast cancer between 2007 and 2018 at Sahlgrenska University Hospital (Gothenburg, Sweden), 414 patients had previously been diagnosed with other primary malignancies.^5^ Clinical data for the 414 patients were retrieved from Sahlgrenska University Hospital (Departments of Clinical Pathology and Oncology) and the Swedish Cancer Registry (National Board of Health and Welfare (Socialstyrelsen)). All tumors were confirmed as primary malignancies, that is, different histopathologic origins and not to be considered as metastases, by a board‐certified pathologist (A.K.) using formalin‐fixed paraffin‐embedded (FFPE) sections stained with hematoxylin and eosin. None of the patients were diagnosed with distant metastasis at the time of diagnosis for either the first or second tumor. Of the 414 patients, women were ≤50 years of age at the time of their BC diagnosis were selected for genomic analysis (n=26) and 25 of these 26 patients had two primary malignancies (including breast cancer). Only one patient had three tumors (patient 25). For each patient, the BC samples were labeled as “A” and all other cancer types as “B” or “C.” The clinicopathologic features of the 26 cases are shown in Table 1. This study was approved by the Regional Ethical Committee in Gothenburg (approval no. 287–15).

**TABLE 1:** Clinicopathologic features for the 26 breast cancer patients (≤50 years of age) with other previous primary malignancies

Patient nr	Cancer 1						Cancer 2													OS time (months)	Family history of breast cancer	*BRCA*‐positive (germline)
	Age at diagnosis	type	TNM	Treatment			Age at diagnosis	TNM	Histopathology	BRE score	Tumor size (mm)	ER&PR (%)	Ki67 (%)	HER2 amplified	Axillary lymph node metastases	Treatment						
				*Surgery*	*Chemo*	*Radiation*										*Surgery*	*Chemo*	*Radiation*	*Other*	200101		
1	28	GYM	Tx Nx Mx	Yes	No	No	50	T1c N0 Mx	IDC	6	11	90 & 100	30–40%	No	No	Yes	No	Yes	Tam	65	No	No
2	41	MM	Tis Nx Mx	Yes	No	No	50	T2 N1 Mx	IDC	7	15	90	<10	No	No	Yes	No	Yes	Tam	71	No	No
3	39	MM	T1a Nx Mx	Yes	No	No	40	T1b N1 Mx	IDC	6	31	100 & 100	10	No	Yes	Yes	Yes	Yes	Tam	94	No	No
4	36	TYM	T3 N0 Mx	Yes	No	No	36	T2 N0 Mx	IDC	5	22	100 & 100	15	No	No	Yes	No	Yes	Tam	65	No	No
5	33	MM	Tis Nx Mx	Yes	No	No	44	T2 N1 Mx	IDC	7	22	100 & 100	5	No	Yes	Yes	Yes(n)	Yes	Tam	63	Yes	C457A>C, unknown variant
6	47	GIM	T3a N2b Mx	Yes	Yes	No	48	T3 N1 Mx	ILC	7	63	50 & 0	5	No	Yes	Yes	Yes(n)	Yes	Tam	62	Yes	No
7	35	MM	T1a Nx Mx	*	*	*	48	Tis N0 Mx	DCIS	*	61	*	*	*	No	No	No	No	No	62	No	No
8	21	GYM	Tx Nx Mx	Yes	No	No	48	T2 N0 Mx	IDC	9	26	100 & 80	40	No	No	Yes	Yes	No	AI	75	No	No
9	28	MM	Tis Nx Mx	Yes	No	No	49	T2 N1 Mx	IDC	6	29	0 & 0	90	No	Yes	Yes	Yes	No	No	17	No	No
10	44	HM	*	No	Yes	No	44	Tis N0 Mx	DCIS	*	76	*	*	*	No	Yes	No	No	No	87	No	No
11	20	Sarcoma	Tx Nx Mx	Yes	No	No	48	T1b N0 Mx	ITC	4	7	90 & 100	5	No	No	Yes	No	Yes	No	107	No	No
12	38	OM	Tx N1 Mx	Yes	No	Yes	39	T1c N0 Mx	IDC	8	19	0 & 0	60	No	No	Yes	No	No	No	61	No	No
13	32	TYM	T4a Nx Mx	Yes	No	No	47	T2 N2a Mx	IDC	8	28	70 & 0	30	Yes	Yes	Yes	Yes	Yes	Tam+Trast	105	No	No
14	43	MM	Tis Nx Mx	Yes	No	No	49	T3 N1 Mx	ILC	6	62	100 & 100	10	No	Yes	Yes	Yes	Yes	Tam	99	No	No
15	40	MM	T2b N2 M1	Yes	No	No	42	T1c N0 Mx	IDC	5	14	90 & 70	30	No	No	Yes	No	Yes	Tam	118	No	No
16	39	MM	Tis Nx Mx	Yes	No	No	44	T3 N2a Mx	ITC	4	66	100 & 100	9	No	Yes	Yes	No	No	Tam	110	No	No
17	42	GYM	*	Yes	Yes	No	48	T2 N1 Mx	IDC	5	30	100 & 100	5	No	Yes	Yes	Yes	Yes	AI	132	No	No
18	24	OM	Tx N1 Mx	*	Yes	No	43	T1c N0 Mx	ITC	4	13	100 & 40	10	No	No	Yes	No	No	No	126	No	No
19	34	MM	T1a Nx Mx	Yes	No	No	44	T2 N0 Mx	ITLC	5	26	100 & 100	10	No	No	Yes	No	Yes	Tam	143	No	No
20	29	HM	*	No	Yes	No	43	T1c N1c Mx	IDC	9	18	0 & 0	60	Yes	Yes	Yes	Yes	Yes	Trast	148	No	No
21	42	MM	T3 Nx M1	Yes	No	No	44	T2 N0 Mx	IMC	9	41	0 & 0	50	No	No	Yes	Yes	Yes	No	48	No	No
22	36	MM	T1a Nx Mx	Yes	No	No	50	T1c N0 Mx	IDC	8	19	100 & 100	30	No	No	Yes	Yes	Yes	Tam+AI	62	No	No
23	25	HM	*	No	No	Yes	45	T3 N2a Mx	IDC	9	51	100 & 50	26	No	Yes	Yes	Yes	Yes	Tam+AI+ev PARP?	48	Yes	3171ins5, founder mutation
24	22	HM	*	No	Yes	Yes	42	T1c N0 Mx	IDC	7	13	0 & 0	20	Yes	No	Yes	Yes	No	Tam+Trast	45	Yes	No
25	31	GYM	T1A1 Nx Mx	Yes	No	No	37	T2 N0 Mx	IDC	9	30	0 & 0	90	No	No	Yes	Yes	No	No	43	Yes	No
26	32	GYM	Tis Nx Mx	Yes	No	No	42	T1b N0 Mx	IDC	7	25	100 & 100	8	No	No	Yes	No	Yes	Tam	41	No	No

Abbreviation: BC, breast cancer; GIM, gastrointestinal malignancies; GYM, gynecological malignancies; HM, hematological malignancies; MM, malignant melanoma; OM, oral cavity malignancies; TM, thyroid malignancies

* no data available; (*n*), neoadjuvant treatment; DCIS, ductal carcinoma in situ; IDC, invasive ductal carcinoma; IMC, invasive medullary carcinoma; ILC, invasive lobular carcinoma; ITC, invasive tubular carcinoma; ITLC, invasive tubulo‐lobular carcinoma TMN classification according to Brierley et al.

### 2.2 OncoScan CNV plus assay

Genome‐wide copy number and mutation analysis were performed for 47/53 samples using Affymetrix OncoScan® Arrays according to standard protocols at the Array and Analysis Facility (Uppsala University, Uppsala, Sweden). The OncoScan somatic mutation panel consisted of 64 mutations in nine genes (*BRAF*, *EGFR*, *IDH1* and *2*, *KRAS*, *NRAS*, *PIK3CA*, *PTEN*, and *TP53*). In brief, genomic DNA was extracted from two to three 10 µm FFPE sections for the 53 tumor samples using the AllPrep DNA/RNA FFPE kit (Qiagen) according to the manufacturer's instructions. The DNA concentration was determined with the Qubit Fluorometer (Life Technologies) and 80 ng genomic DNA subsequently used in the OncoScan assay. Five samples (1B, 2A/2B, and 16A/16B) were excluded due to low DNA concentration or lack of DNA amplification. Only pairwise samples (A and B samples) were included in the analysis; Sample 25C was excluded.

#### 2.2.1 DNA copy number and mutation analysis

Normalization, segmentation, and quality control of the raw intensity (CEL) files were performed using the Chromosome Analysis Suite (ChAS, v4.1.0.90(r294000)) from Thermo Fisher Scientific with the hg19 genome assembly and NA33 FFPE analysis workflow. Mutations identified in the OncoScan somatic mutation panel (e.g., missense mutations) and allelic imbalance data (e.g., log_2_ratio, allele difference, BAF, and LOH) were extracted from ChAS. Data for the clinical significance of the identified mutations were retrieved from the dbSNP and ClinVar database (https://www.ncbi.nlm.nih.gov/snp/ and https://www.ncbi.nlm.nih.gov/clinvar/). Genomic profiles (hg19 genome assembly) were generated using the rCGH package (v1.16.0)^27^ in R/Bioconductor (v3.6.1) with log_2_ratio thresholds set to +0.3 for gains and −0.3 for losses. Further analysis to compare genomic profiles for the two tumors in the same patient or between different cancer types was performed using Nexus Copy Number (BioDiscovery v8.1) with normalized OSCHP files and a 25% differential threshold between groups (*p* < 0.05). The OSCHP files were loaded with the Affymetrix OSCHP‐TuScan algorithm for the hg19 genome assembly. Genomic regions covered entirely by previously reported copy number variations in the human genome were removed.^28^ Descriptive statistics (mean  ± standard error of the mean (SEM) and range) for the number of genetic alterations in each tumor were calculated using Microsoft Excel (v16.16.27). Box plots were constructed using the ggplot2 (v3.3.1) and ggpubr (v0.3.0) ^29^, ^30^ R packages with the Wilcoxon test.

#### 2.2.2 Similarity and clonality analysis

To evaluate whether the genomic profiles for tumors from the same patient were similar, hierarchical clustering, calculation of the Similarity Index (SI), and clonality testing were performed. First, hierarchical clustering of unsegmented CNA or LOH data was performed using the Euclidean distance metric and Ward's minimum variance method (Ward.D2) with the ggdendro R package (v0.1.22).^31^ Tumors from the same patient were considered to be similar if they clustered together in the terminal branch of the dendrogram. Then, the SI was calculated as described elsewhere using unsegmented data.^32^ In brief, SI was calculated by determining unique, shared, and opposite CNA or LOH changes between tumor pairs using CNA log_2_ratio thresholds set to +0.3 for gains and −0.3 for losses and LOH thresholds set to 0 for normal DNA segments and 1 for LOH. Tumor pairs with Benjamini–Hochberg adjusted *p* < 0.05 in the SI analysis were considered to be similar. Last, clonality testing with the Clonality R package (v1.34.0) was performed to determine whether tumors from the same patient were clonal or independent entities.^33^ Tumor pairs with *p* < 0.05 in the clonality analysis were considered to be clonal.

#### 2.2.3 Genetic instability analysis

To identify genetically unstable tumors, three analyses were performed with segmented CNA data, that is, genetic instability index (GII), complex arm‐wise aberration index (CAAI), and chromothripsis‐like pattern (CTLP) detection. GII was calculated as described elsewhere using CNA log_2_ratio thresholds set to +0.3 for gains and −0.3 for losses; genomic instability was defined as GII >0.2.^34^, ^35^, ^36^ CAAI detects complex focal rearrangements in the genome containing narrow regions of high copy number gain; CAAI‐positivity was defined as tumors with CAAI ≥0.5.^37^, ^38^, ^39^ For CTLP, CNA data were segmented using the DNAcopy package (v1.60.0) in R,^40^ followed by CTLP detection using the web‐based CTLPScanner (http://47.88.3.162/CTLPScanner/) with default settings (Genome assembly: GRCh37/hg19; Copy number status change times: ≥20; Log10 of likelihood ratio ≥8; Minimum segment size (Kb): 10; Signal distance between adjacent segments: 0.3; Genomic gains ≥0.3; Genomic losses ≤−0.3.^41^

## 3 RESULTS

### 3.1 Selection of young patients (≤50 years) with other primary malignancies before breast cancer

We recently described a cohort of 414 patients diagnosed with one or more primary malignancies before BC (diagnosed with BC between 2007 and 2018) at Sahlgrenska University Hospital in Gothenburg, Sweden.^5^ Of the 414 patients, 26 women were ≤50 years of age at the time of their breast cancer diagnosis. Interestingly, 1/26 patient was diagnosed with three different malignancies (two different gynecological malignancies and breast cancer). Therefore, the 26 patients corresponded to 53 tumors (Table 1). The mean size of the detected invasive breast carcinomas was 28.3 mm and the average BRE score was 6.6. For 11 of the 26 patients, the metastatic spread of breast cancer was detected in the axillary lymph nodes. Four patients had triple‐negative breast cancer and three patients had HER2‐amplified breast cancer. A detailed analysis of the medical records revealed five patients with a family history of breast cancer (two patients with HM, one patient each with MM, GIM, and GYM), of which two were *BRCA*‐positive (C457A>C, unknown variant; and 3171ins5, founder mutation). The most common OPPM detected in the 26 breast cancer patients were malignant melanoma (MM, *n* = 11), gynecological malignancies (GYM, *n* = 5), and hematological malignancies (HM, *n* = 4). Other OPPMs included gastrointestinal malignancies (GIM, *n* = 1), thyroid malignancies (TM, *n* = 2), oral cavity malignancies (OM, *n* = 2), and sarcoma (*n* = 1). Due to loss of DNA, only nine of the MM and four of the GYM were analyzed.

### 3.2 Genetic alterations more common in BC and GYM

**FIGURE 1:**
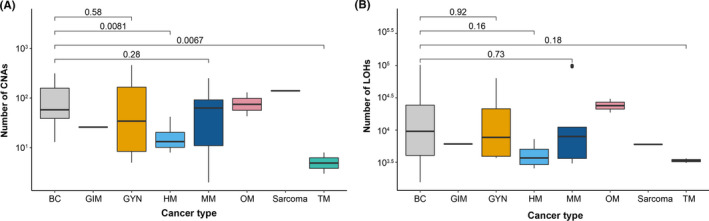
Boxplots depicting the number of detected (A) CNAs and (B) LOH in the different cancer types. The Wilcoxon test was used to calculate statistically significant differences in BC versus previous primary malignancies (GYM, HM, MM, and TM). GIM, OM, and sarcoma were excluded from the statistical analyses due to too few samples. *P* < 0.05 is considered to be statistically significant

To identify DNA copy number alterations (CNA) and somatic mutations in the 53 patient samples, genome‐wide Affymetrix OncoScan® Arrays were used. However, six samples were removed from further analysis due to technical reasons. Genetic alterations (DNA low‐level gains, heterozygous losses, homozygous deletions, and high‐level amplification) were subsequently detected in all 47 samples, affecting whole chromosomes, chromosome arms, and specific chromosomal regions. The highest number of CNAs was detected in GYM (mean ± SEM, 148 ± 228; range, 5–461), followed by BC (mean ± SEM, 100 ± 157; range, 0–314) and OM (mean ± SEM, 86 ± 44; range, 43–130). Overall, BC was shown to harbor significantly more CNAs than HM (*p* = 0.0081) and TM (*p* = 0.0067; Figure 1A). In contrast, relatively few CNAs were shown in the two TM samples (mean ± SEM, 5.5 ± 2.5; range, 3–8). As expected, the CNAs detected in BC were primarily low‐level gains (mean ± SEM, 51 ± 90; range, 0–179), with fewer regions of high‐level amplification (mean ± SEM, 14 ± 84; range, 0–169), heterozygous loss (mean ± SEM, 25 ± 60; range 0–1372), and homozygous deletion (mean ± SEM, 1.0 ± 6.5; range, 0–13). GYM also showed more low‐level gain (mean ± SEM, 98 ± 154; range, 3–310) than heterozygous loss (mean ± SEM, 31 ± 39; range, 0–78). Moreover, high‐level amplifications were more common in both BC (mean ± SEM, 14 ± 84; range, 0–169) and GYM (mean ± SEM, 19 ± 36; range, 0–73) compared to MM (mean ± SEM, 0.1 ± 0.5; range, 0–1).

In agreement with previous studies,^42^, ^43^, ^44^, ^45^, ^46^, ^47^ genetic alterations in BC were frequently detected on chromosome 1 (mean ± SEM, 1.8 ± 11; range 0–22) of which 66% were low‐level gains (mean ± SEM, 4.9 ± 9; range, 0–18), followed by chromosome 16 (mean ± SEM, 1.5 ± 10; range, 0–20) of which 52% consisted of low‐level gains (mean ± SEM, 3 ± 10; range, 0–20) and chromosome 17 (mean ± SEM, 1.5 ± 14; range, 0–28) of which 35% consisted of high‐level amplifications (mean ± SEM, 2.1 ± 14; range, 0–28) and 35% of heterozygous losses (mean ± SEM, 2.1 ± 3; range, 0–6; Table S1). More specifically, low‐level gains frequently detected in BC spanned chromosomal regions 1q, 8q, 10p, 14q, 16p, and 20q, while heterozygous loss was identified on 4p, 8p, 11q, 16q, 17p, and 22q. For GYM, genetic alterations were detected on all chromosomes, equally distributed between gains and losses where chromosomes 2 (mean ± SEM, 2.1 ± 13; range, 0–26) and 10 (mean ± SEM, 2 ± 10; range, 0–20) were most prevalent. However, CNAs in MM were common on chromosome 3 (mean ± SEM, 1.4 ± 7.5; range, 0–15), 8 (mean ± SEM, 1.1 ± 6.5; range, 0–13), and 19 (mean ± SEM, 1 ± 10.5; range, 0–21). The number of CNAs detected on each chromosome is presented in Table S1.

For 6/9 BCs from patients previously diagnosed with MM, low‐level gains were identified on chromosome 1q, while DNA losses affected chromosomes 6 and 17p. Furthermore, low‐level gain was frequently shown on chromosomes 3p and 14p in MM. Common DNA copy number alterations were detected on chromosomes 9, 10, 14, 18, and 22 in BC and corresponding GYM, the majority of which were low‐level gains. However, two of the four pairwise GYM and BC shared high‐level amplification on chromosome 22. Common DNA losses on chromosomes 8 and 11 were seen in three BC and corresponding HM (75%). Low‐level gains on chromosomes 2, 8, 13, and 16 were commonly seen in BC and corresponding OM. However, no common DNA alterations were detected in BC and corresponding TM.

### 3.3 Loss‐of‐heterozygosity (LOH) more common in BC, MM, and GYM

The OncoScan data were then analyzed to identify LOH in individual SNP markers. Similar to the detected DNA copy number alterations, LOH patterns in the different tumors were complex with every chromosome displaying LOH at least once. More specifically, LOH affected whole chromosomes, chromosome arms, specific chromosome regions, and individual LOH markers. The highest number of LOH events detected in the 47 tumors were located on chromosomes 1, 2, 5, 6, 11, and 17. Furthermore, the highest number of LOH events were seen in BC followed by MM and GYM. In contrast, TM presented the lowest number of LOH events (Figure 1B). In BC, chromosomes 1, 4, 6, 9, 13, 18, and 20 displayed the highest number of LOH events, whereas chromosomes 2, 3, 5, 9, and 20 were frequently affected by LOH in GYM and chromosomes 4, 9, and 11 in MM. Intriguingly, LOH events on chromosome 6 were common in BC, OM, HM, and sarcoma. However, OM, TM, and GIM were also found to be affected by LOH on chromosomes 6, 7, and 12 (OM), chromosomes 3, 11, and 12 (TM), and chromosome 10 (GIM). The recurrent LOH regions in the tumor samples are presented in Table S2.

### 3.4 Mutations in *TP53* prevalent in BC, MM, and HM

**FIGURE 2:**
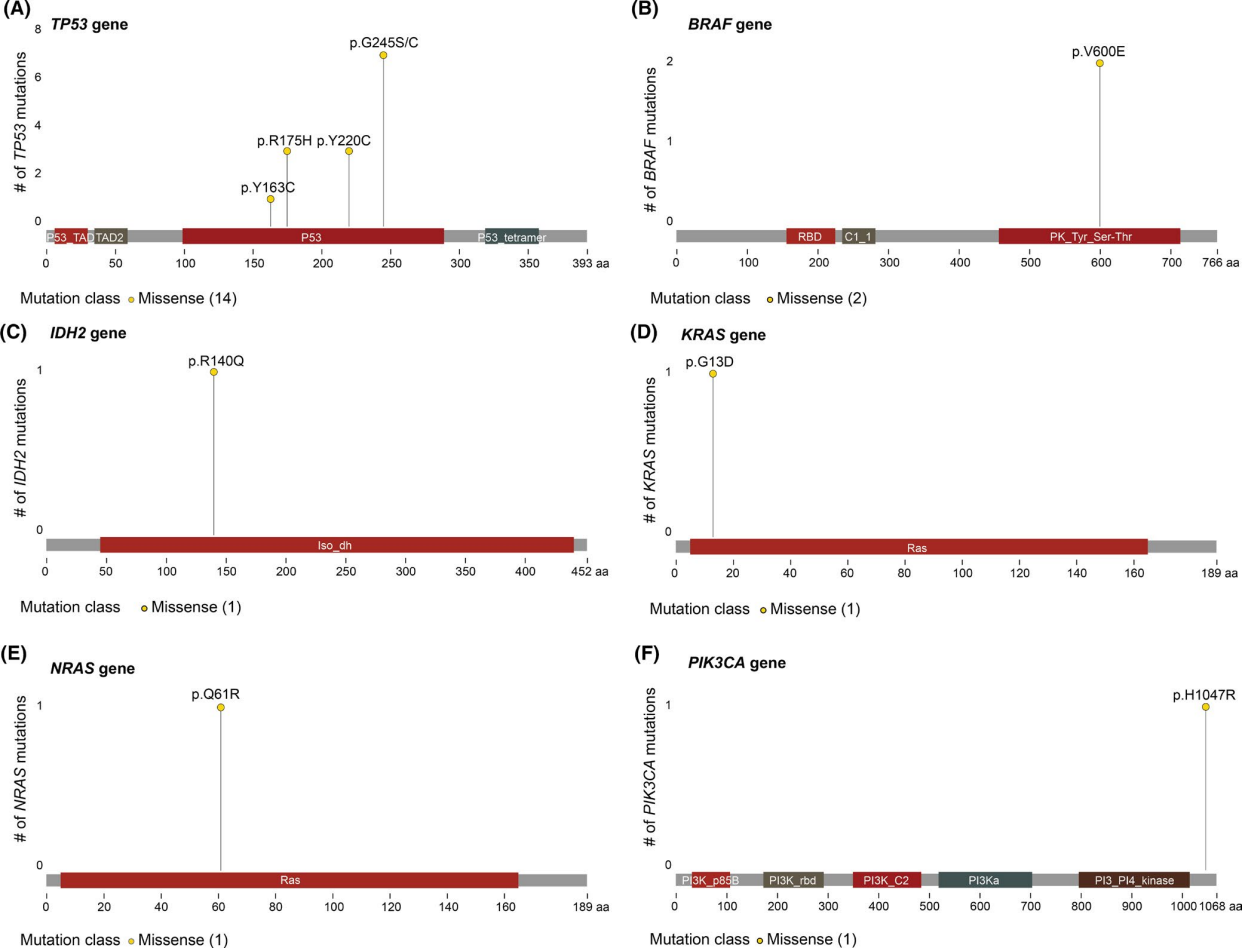
Lollipop plots depicting the number and type of mutations spanning the (A) TP53, (B) *BRAF*, (C) *IDH2*, (D) *KRAS*, (E) *NRAS*, and (F) *PIK3CA* genes according to the OncoScan somatic mutation panel

The OncoScan somatic mutation panel was then used to analyze 64 specific mutations in nine cancer‐related genes (*BRAF*, *KRAS*, *EGFR*, *IDH1*, *IDH2*, *PTEN*, *PIK3CA*, *NRAS*, and *TP53*). Two patients (patients 5A and 23A) with a family history of breast cancer previously tested positive for *BRCA* mutations. Consequently, the OncoScan array detected mutations in the *TP53* gene for 14/47 tumors, of which 8 were identified in BC (33% of the BCs), 2 in MM (22% of the group of MM), and 2 in HM (50% of the HM; Figure 2 and Table S3). Other mutations were also detected, but to a lesser extent, that is *KRAS* (n=1), *NRAS* (n=1), *IDH2* (*n* = 2), *BRAF* (*n* = 2), and *PIKCA3* (*n* = 1). Interestingly, the same mutation (*TP53*:p.Y220C:c.659A>G) was only found in one tumor pair (BC and MM) from the same patient (patient 21). Reported clinical significance of SNP is presented in Table S3.

### 3.5 Genomic profiling reveals similarity between tumor pairs

**FIGURE 3:**
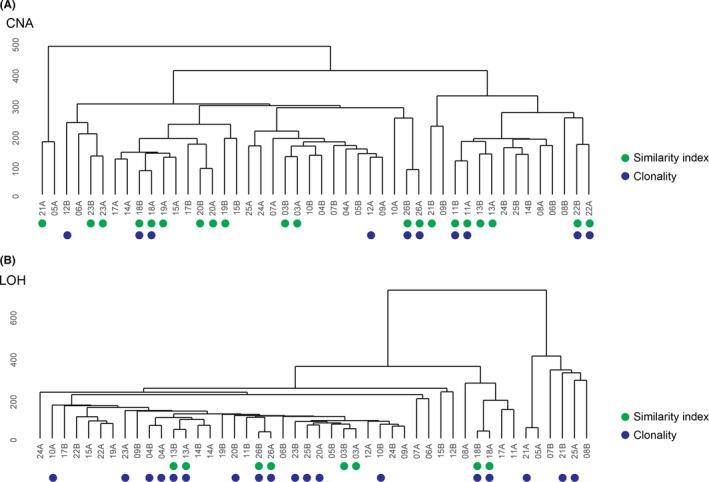
The concordance between hierarchical clustering, similarity index, and clonality testing using (A) CNA and (B) LOH data from OncoScan. Similarity in the pairwise tumors was found in all three analyses for patients 18 and 26 using both CNA and LOH data

**FIGURE 4:**
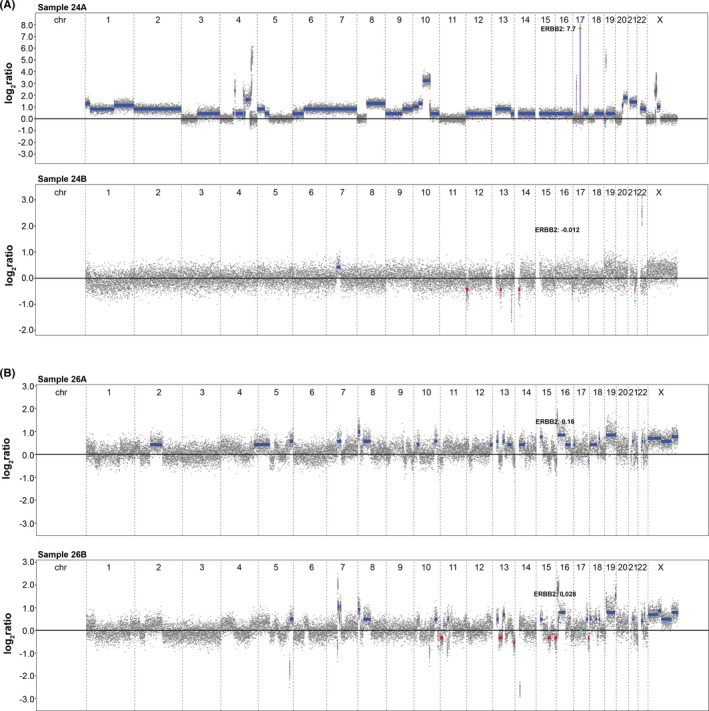
Genomic profiles for the tumor pairs for patients (A) 24 (BC and HM) and (B) 26 (BC and GYM). The copy number profiles for patient 24 are drastically different, while the samples from patient 26 were similar. The location of the *ERBB2* gene is indicated on chromosome (chr) 17 with the log_2_ratio value. Blue bars indicate DNA gain (log*2*ratio threshold +0.3) and red bars depict DNA loss (log*2*ratio threshold −0.3)

To evaluate whether the genomic profiles (CNA and LOH) for tumors from the same patient were similar, three different analyses (hierarchical clustering, Similarity Index (SI), and clonality testing) were performed using the OncoScan data. Hierarchical clustering and SI were concordant for tumor pairs from eight (patients 03, 11, 13, 18, 20, 22, 23, and 26) and four ^13^, ^18^, ^26^ patients, which were classified as genomically similar using the CNA and LOH data, respectively (Figure 3). In contrast, clonality testing was only concordant with clustering and SI for four (patients 11, 18, 22, and 26) and three (patients 13, 18, and 26) patients. Nevertheless, hierarchical clustering also identified other tumor pairs (patients 04 and 08 for CNA, and 04, 14, 21, and 22 for LOH) as closely related, but with less overlap between the genetic alterations. No significant similarity was detected in patients 04–10, 12, 14–17, 21, and 24–25 according to SI. Moreover, no obvious patterns were found regarding which OPPMs and BC were most similar. However, several BCs clustered together followed by MM and TM. Interestingly, clonality testing showed that genetic alterations on chromosomes 1 (*n* = 3), 3 (*n* = 5), 16 (*n* = 3), and 19 (*n* = 3) were concordant in both tumors in patients 06, 07, 08, 09, 11, 12, 14, 18, 19, 21, 22, 25, and 26 (Figure 4).

### 3.6 High levels of genomic instability in BC and OPPMs

Last, the identified DNA copy number alterations were used to determine genetic instability in each sample with the genetic instability index (GII), complex arm‐wise aberration index (CAAI index), and chromothripsis‐like patterns (CTLP). GII revealed that genomic instability was more prevalent in OPPMs (08B, 09B, 12B, 19B, 21B, 22B, and 26B) than BC (05A, 06A, 10A, and 21A). Average CAAI scores (mean and maximum) were calculated and presented in Table S4. All 47 tumors were classified by the CAAI algorithm as unstable. BC (*n* = 14) was among the majority (n=19) of the tumors with the highest CAAI (range 0–8.2). Adenocarcinoma in situ cervix had the highest CAAI (mean 8.2), but CTLP was mostly seen in BC (*n* = 4). More specifically, regions of chromothripsis‐like patterns spanned chromosomes 15 and 17 in sample 07A, sample 08A on chromosomes 10 and 11, sample 10A on chromosome 17, and sample 24A on chromosomes 4 and 10. Only one OPPM (sarcoma, sample 11B) had chromothripsis‐like patterns, which spanned chromosome 19.

## 4 DISCUSSION

In the present study, genome‐wide profiling was used to identify common genetic alterations in primary tumors (BC and OPPM) from the same patient, thereby enabling us to pinpoint potential biomarkers for MPMs. Although BC was revealed to have the highest number of DNA copy number alterations, all 47 tumors were classified as genetically unstable. Moreover, mutations in *TP53* were prevalent, especially in BC, MM, and HM. Further analysis of the genomic profiles revealed eight tumor pairs with common genetic alterations. Nevertheless, tumor samples from the same cancer type frequently clustered together (BC, MM, and TM), indicating more genetic similarity within a cancer type than between tumor pairs from the same patient.

Thus far, few studies have explored genetic alterations in MPMs, in particular in patients with OPPMs diagnosed before breast cancer. A recent case report performed next‐generation sequencing analysis on both the four different tumors and blood from a patient.^48^ However, no common genetic alterations were found in the four tumor samples. Over a decade ago, Mellemkjaer et al and Raymond et al described MPMs that arose after a breast cancer diagnosis, suggesting that BC treatment itself could have contributed to the development of these multiple primary malignancies.^3^, ^6^, ^49^ The majority of the BCs showed unfavorable histopathologic diagnoses with a high proportion of lymph node‐positive patients. In previous studies, several factors have been linked to the poor prognosis associated with developing breast cancer at a young age, including large tumor size at diagnosis, mitotic rate, high tumor grade, lymph node‐positive status, elevated HER2 expression, and low estrogen and progesterone receptor expression.^50^, ^51^ Here, we chose to analyze tumors from young women (≤50 years) with several malignancies to identify genetic alterations associated with genetic susceptibility and not lifestyle factors. Luciani et al reported that elderly patients are more prone to developing MPMs compared to younger patients due to the accumulation of genetic alterations over time, aging, and time of life expectancy.^52^ Therefore, MPMs are expected to be influenced by increasing age.^1^, ^8^, ^52^ However, young women with breast cancer generally have a poorer outcome compared to elderly patients.^50^ Since the clinical outcome is more unfavorable for young patients with breast cancer and OPPMs, it is important to identify genetic alterations associated with the risk of developing future cancers.

The highest number of somatic genetic alterations occurred in the analyzed breast tumor specimens compared with the different OPPMs. The role of somatic CNAs in cancer progression was recently reported in several cancer types, thereby showing that breast carcinoma had the highest number of driver genes.^53^ Breast tumors frequently contain more genetic alterations than other tumor types (our own unpublished data). Here, we showed that eight pairwise primary tumors had common genetic alterations, indicating that these samples share common driver genes. The remaining tumor pairs showed different genetic alterations, with differing tumor biology and driver genes. Furthermore, the homology within the group of breast tumors was more similar compared to the pairwise tumors or other cancer types. Unfortunately, no specific genetic alterations were found between breast cancer and OPPMs. Genetic alterations on chromosomes 1, 11, and 17 were frequently detected in BC specimens, which could indicate that these changes are breast cancer‐specific. LOH in chromosomes 1, 11, and 17 have previously been reported as indicators of genetic instability and may serve as prognostic factors of poor outcome in breast cancer patients.^54^, ^55^ Genomic instability is a marker of increased risk of developing other primary malignancies as a result of altered DNA damage repair mechanisms.^56^, ^57^, ^58^, ^59^ All of the analyzed tumors were classified as genomically unstable according to the GII and CAAI analyses. Furthermore, another indication of genomic instability in the breast tumors was seen as CLTPs on chromosomes 10 and 17. In contrast, only one OPPM was found to contain CTLPs.

Previous studies have described different somatic mutations in BC.^60^, ^61^, ^62^ In addition, several studies state that CNAs are a common feature of genetic instability in breast carcinomas.^47^, ^63^ Nevertheless, no studies to date have compared somatic mutations in BC with OPPMs. Here, the OncoScan mutation panel was used to evaluated potential somatic mutations in the analyzed specimens. However, this mutation panel only consists of 64 mutations covering nine genes, which provides relatively low coverage of the mutational landscape. Genomic instability is manifested by an increased rate of somatic mutations,^64^ which unfortunately was not possible to explore using this platform. Mutations in the *TP53* gene were detected in 14 of the 47 tumors, of which the majority (*n* = 8) were seen in BC. All breast tumors with *TP53* mutations were large in size (mean 39.2 mm), but three were from lymph node‐positive patients. Previous reports showed a strong association and linear relationship between tumor size, lymph node positivity, and the frequency of *TP53* mutations, which is in agreement with our findings showing that *TP53* mutations are generally associated with an advanced and aggressive tumor phenotype.^65^, ^66^ The *TP53* gene is involved in cell survival, genomic integrity (instability and repair), apoptosis, and proliferation.^65^ Other genetic alterations in *BRAF*, *IDH2*, *KRAS*, *NRAS*, *PIK3CA*, and *TP53* were also reported. Interestingly, data from the dbSNP (https://www.ncbi.nlm.nih.gov/snp and https://cancer.sanger.ac.uk/cosmic) database demonstrate that these mutations play a clinically significant role in the development of other malignancies. In a recent study concerning somatic mutations in young patients with breast and serous ovarian cancer, Encinas et al report that certain mutations are linked to age.^8^, ^9^ Consequently, the incidence of *TP53* somatic mutations tends to increase in the elderly.^8^, ^9^, ^65^ In the present study, we identified nine missense mutations that have been reported to be potentially pathogenic in other malignancies (e.g., gastrointestinal organs, hematopoietic system, brain, and thyroid glandule). Therefore, these SNPs may play a key role in the development of different types of cancers.

The present study has two major strengths, that is, the inclusion of young patients with several primary malignancies in the study cohort and investigating genetic alterations in OPPMs diagnosed before BC. At young ages, the accumulation of genetic alterations in key cancer‐related genes is less dependent on time‐related exposure to environmental factors.^1^, ^8^, ^9^, ^52^ It is also a fact that different population groups have different predispositions for specific cancers. In the present investigation, the analyzed tumors come from a relatively homogeneous population in Sweden which is ideal for the identification of genetic alterations associated with patient's genetic susceptibility. This is also the first study, to the best of our knowledge, to assess genetic alterations in OPPMs before a breast cancer diagnosis. However, our study also has some limitations, for example, the relatively restricted number of patients and the exclusion of six samples due to technical issues. Hence, the statistical analyses were limited by a low number of samples in each OPPM group. More studies with larger patient cohorts are therefore warranted. Unfortunately, only FFPE samples were available, which frequently have poorer DNA quality than frozen samples. However, the genomic profiles were relatively good for the analyzed samples. Last, though the OncoScan array can identify genome‐wide CNAs, LOH, and CTLPs, the mutation panel is limited to 64 somatic mutations in specific genes. In future studies, it would be more appropriate to used high‐resolution technologies such as genome‐wide sequencing on all primary malignancies and corresponding normal tissue sample and whole‐exome sequencing of the primary malignancies and patient blood samples to identify germline mutations. Therefore, it would be possible to identify common alterations important for tumor development, that is, driver mutations.^60^, ^61^, ^62^ Multiple malignancies involve a complex set of common, recurrent within the same type of tumors, and less frequent within the tumor pairs genomic abnormalities. The aim is to develop a model for the initiation and progression of multiple malignancies. The identification of genetic alterations detected in this study may highlight the potential sites for genomic regions susceptible to multiple malignancies initiation and progression.

In the past, follow‐up programs have varied for breast cancer patients. Nowadays, breast cancer patients in Sweden have only 1 year of clinical follow‐up before the patient is referred back to the general mammography screening program. However, previous studies highlight the importance of establishing tailored follow‐up programs and awareness that some cancer patients have an elevated risk of developing additional primary malignancies.^4^, ^5^, ^6^, ^49^, ^67^ Lee et al state the importance of establishing guidelines for improving prognosis and quality of life in breast cancer patients.^67^ To be able to improve cancer outcomes, possible new malignancies should be investigated as the first sign of symptoms. According to Raymond et al, the interval between different primary malignancies in patients aged 30–39 was 11.4 years. Therefore, Raymond et al proposed that breast cancer survivors should be advised of their increased risk of developing certain cancers in their lifetime. This could influence the patient's lifestyle choices related to smoking, exercise, weight, exposure to UV radiation, etc.^3^ Bleyer et al also emphasized the importance of tailoring treatment strategies in different age groups, as tumor biology depends on the age group.^10^ However, there may be a way to predict the development of additional primary malignancies, or at least be prepared for the risk of their development. Spratt et al described the time labeling index that suggests that one and the same mutation could contribute to different metachronous malignancies due to differences in doubling time for each independent tumor.^58^, ^59^

Taken together, young patients with OPPMs and BC should be informed of the risk of developing other malignancies. In case of symptoms, the patient should be promptly examined to rule out additional malignancies and follow‐up programs tailored to the patient's respective malignancies and associated somatic mutations. However, further studies are warranted to investigate the clinical implications of genetic alterations for the diagnosis, treatment,^21^ and follow‐up programs for MPMs.

## CONFLICTS OF INTERESTS

The authors declare that they have no competing interests.

## AUTHOR CONTRIBUTIONS

JN, KH, TP, PK, EFA, ZE, AK were responsible for the overall study concept. KH, TP, and JN were responsible for the design of experiments. JN, AK, ZE collected the clinical data. JN, KH, and TP contributed to the statistical analyses. JN, TP, KH analyzed the data, performed the statistical analyses, and wrote the manuscript. All authors reviewed, edited, and approved the final manuscript.

## ETHICAL STATEMENT

Ethics approval All procedures were performed in accordance with the Declaration of Helsinki and approved by the Regional Ethical Review Board (Gothenburg, Sweden; Case Numbers 287–15, T967‐17, T1033‐18, and T2019‐04294).

## Supporting information

Table S1

Table S2

Table S3

Table S4

## Data Availability

The datasets used and analyzed during the current study are available from the corresponding author on reasonable request. All microarray data discussed in this publication are accessible through the NATIONAL CENTER for Biotechnology Information (NCBI) Gene Expression Omnibus (GEO accession number GSE165240).
