# Supplementary material for: Genetic alterations associated with multiple primary malignancies
Source: Cancer Med. 2021 May 31;10(13):4465–77. doi: 10.1002/cam4.3975 (PMC8267160; doi:10.1002/cam4.3975)
Supplement: Supplementary file 1 — Table S1 [file CAM4-10-4465-s003.docx]

Supplementary Table 1. The number of CNAs detected on each chromosome in the different cancer types

|  | BC | |  |  |  | MM | |  |  |  | GYN | |  |  |  | GIM | |  |  |  | THYR | |  |  |  | ORAL | |  |  |  | HEMA | |  |  | SARCOMA | | |
| --- | --- | --- | --- | --- | --- | --- | --- | --- | --- | --- | --- | --- | --- | --- | --- | --- | --- | --- | --- | --- | --- | --- | --- | --- | --- | --- | --- | --- | --- | --- | --- | --- | --- | --- | --- | --- | --- |
| Chrom | *HD HL* | *LLG* | *HLA* |  | *HD* | *HL* | *LLG* | *HLA* |  | *HD* | *HL* | *LLG* | *HLA* |  | *HD* | *HL* | *LLG* | *HLA* |  | *HD* | *HL* | *LLG* | *HLA* |  | *HD* | *HL* | *LLG* | *HLA* |  | *HD* | *HL* | *LLG* | *HLA* | *HD* | *HL* | *LLG* | *HLA* |
| 1 | 0.04 1.08 | 4.92 | 1.38 |  | 0.11 | 1.67 | 2 | 0 |  | 0.5 | 3 | 2.5 | 0.75 |  | 0 | 1 | 1 | 0 |  | 0 | 0 | 0 | 0 |  | 0 | 0.5 | 0.8 | 0.2 |  | 0 | 0.25 | 0.75 | 0 | 0 | 3 | 2 | 0 |
| 2 | 0.04 1.17 | 2.54 | 0.29 |  | 0 | 0.89 | 2.56 | 0 |  | 0 | 1.75 | 6.5 | 0.25 |  | 0 | 0 | 1 | 0 |  | 0 | 0 | 0 | 0 |  | 0 | 1.5 | 1.6 | 1 |  | 0 | 0.5 | 0 | 0 | 0 | 3 | 3 | 0 |
| 3 | 0.04 1.21 | 2.79 | 0.21 |  | 0 | 1.33 | 4.44 | 0 |  | 0 | 1 | 1 | 0.75 |  | 0 | 0 | 1 | 0 |  | 0 | 0.5 | 1 | 0 |  | 0 | 0 | 1.2 | 0.6 |  | 0 | 0.25 | 0.5 | 0 | 0 | 1 | 8 | 0 |
| 4 | 0 1.3 | 1.67 | 0.67 |  | 0 | 0.78 | 2.89 | 0 |  | 0 | 0.25 | 3.5 | 0.25 |  | 0 | 0 | 1 | 0 |  | 0 | 0 | 0 | 0 |  | 0 | 0 | 0.4 | 0.4 |  | 0 | 0 | 0 | 0 | 0 | 1 | 10 | 0 |
| 5 | 0.04 1.21 | 1.63 | 0.08 |  | 0 | 1.33 | 1.89 | 0 |  | 0 | 0.25 | 3.25 | 0.75 |  | 0 | 1 | 0 | 0 |  | 0 | 0 | 0 | 0 |  | 0 | 2.5 | 0.8 | 0.8 |  | 0 | 0.25 | 0 | 0 | 0 | 0 | 0 | 0 |
| 6 | 0.08 1.63 | 2.54 | 0.46 |  | 0 | 1.22 | 1.56 | 0 |  | 0 | 1.5 | 4 | 0.25 |  | 0 | 0 | 0 | 0 |  | 0 | 0 | 0 | 0 |  | 0 | 4 | 1.6 | 1.6 |  | 0 | 1 | 0 | 0 | 0 | 6 | 1 | 0 |
| 7 | 0 0.61 | 2.5 | 1.33 |  | 0 | 0.67 | 2.22 | 0 |  | 0 | 1.5 | 4.25 | 1.25 |  | 0 | 0 | 0 | 0 |  | 0 | 0 | 0 | 0 |  | 0 | 5 | 1.8 | 1.6 |  | 0 | 0 | 0.25 | 0 | 0 | 0 | 1 | 0 |
| 8 | 0.08 1.5 | 3.42 | 0.75 |  | 0 | 1.56 | 2.89 | 0 |  | 0 | 1 | 4.75 | 1.5 |  | 0 | 1 | 0 | 0 |  | 0 | 0 | 0 | 0 |  | 0 | 2 | 1.4 | 0.8 |  | 0 | 1 | 0.25 | 0 | 0 | 2 | 7 | 0 |
| 9 | 0.13 0.75 | 1.67 | 0.04 |  | 0 | 1.11 | 2 | 0 |  | 0 | 0.75 | 2.25 | 0 |  | 0 | 0 | 0 | 0 |  | 0 | 0 | 0.5 | 0 |  | 0 | 2.5 | 2 | 1.6 |  | 0 | 0 | 0 | 0 | 0 | 1 | 3 | 0 |
| 10 | 0 1.29 | 1.75 | 0.92 |  | 0 | 1.67 | 1.33 | 0 |  | 0 | 1.25 | 5.25 | 1.5 |  | 0 | 4 | 0 | 0 |  | 0 | 0 | 0 | 0 |  | 0 | 1 | 1.2 | 1.2 |  | 0 | 0.25 | 0.25 | 0 | 0 | 4 | 3 | 0 |
| 11 | 0 1.83 | 1.54 | 0.71 |  | 0 | 1.56 | 2.11 | 0 |  | 0 | 1 | 4.5 | 0.5 |  | 0 | 1 | 0 | 0 |  | 0 | 0.5 | 0 | 0 |  | 0 | 0.5 | 0.6 | 0.8 |  | 0 | 0.75 | 0.25 | 0 | 0 | 3 | 1 | 0 |
| 12 | 0 0.67 | 1.63 | 0.71 |  | 0 | 1.22 | 2.44 | 0 |  | 0 | 2 | 1 | 0.5 |  | 0 | 0 | 8 | 0 |  | 0 | 0 | 0 | 0 |  | 0 | 1 | 0.8 | 1.2 |  | 0 | 0.25 | 0.25 | 0 | 0 | 2 | 4 | 0 |
| 13 | 0.04 1.25 | 1.29 | 0.042 |  | 0 | 1.33 | 0.89 | 0 |  | 0 | 0.25 | 1.5 | 0 |  | 0 | 0 | 0 | 0 |  | 0 | 0 | 0 | 0 |  | 0 | 3 | 2.2 | 1.8 |  | 0 | 0.5 | 0.25 | 0 | 0 | 1 | 0 | 0 |
| 14 | 0.13 1.29 | 2.33 | 0.58 |  | 0 | 1.78 | 1.78 | 0 |  | 0 | 1.25 | 5.5 | 0.5 |  | 0 | 0 | 0 | 0 |  | 0 | 0.5 | 0 | 0 |  | 0 | 2 | 1.2 | 1.2 |  | 0.25 | 1.5 | 0.25 | 0 | 0 | 2 | 4 | 0 |
| 15 | 0.04 0.83 | 2.04 | 0.88 |  | 0 | 1.11 | 1.11 | 0 |  | 0 | 0.75 | 1.5 | 0.75 |  | 0 | 0 | 0 | 0 |  | 0 | 0 | 0 | 0 |  | 0 | 1 | 0.8 | 0.8 |  | 0 | 0.25 | 0.5 | 0 | 0 | 0 | 1 | 0 |
| 16 | 0.13 1.75 | 3 | 1.04 |  | 0 | 1 | 3 | 0 |  | 0 | 0.25 | 4.5 | 1.5 |  | 0 | 0 | 0 | 0 |  | 0 | 0.5 | 0 | 0 |  | 0.5 | 2 | 1.8 | 1.4 |  | 0 | 0.25 | 1.5 | 0 | 0 | 0 | 3 | 0 |
| 17 | 0.13 2.13 | 1.79 | 2.13 |  | 0 | 1.33 | 1.56 | 0 |  | 0 | 1 | 3 | 1.5 |  | 0 | 0 | 0 | 0 |  | 0 | 0 | 0 | 0 |  | 0.5 | 3.5 | 2 | 2 |  | 0 | 0.75 | 2.25 | 0.25 | 0 | 2 | 0 | 0 |
| 18 | 0 0.92 | 1.33 | 0.08 |  | 0 | 0.67 | 1.78 | 0 |  | 0 | 1 | 4.75 | 0.5 |  | 0 | 0 | 0 | 0 |  | 0 | 0 | 0 | 0 |  | 0 | 3 | 1.8 | 1.6 |  | 0 | 0 | 0 | 0 | 0 | 1 | 1 | 0 |
| 19 | 0 1.13 | 3.38 | 0.75 |  | 0 | 1 | 3.44 | 0 |  | 0 | 0.25 | 3.75 | 2.75 |  | 0 | 0 | 2 | 0 |  | 0 | 0 | 0 | 0 |  | 0 | 2 | 2.2 | 2.2 |  | 0 | 0.5 | 1.5 | 0 | 0 | 3 | 15 | 0 |
| 20 | 0 0.67 | 1.69 | 0.38 |  | 0 | 0.67 | 2.89 | 0 |  | 0 | 0.5 | 2.25 | 0.5 |  | 0 | 0 | 3 | 0 |  | 0 | 0 | 0.5 | 0 |  | 0 | 1 | 0.8 | 0.8 |  | 0 | 0.25 | 0 | 0 | 0 | 0 | 0 | 0 |
| 21 | 0 0.75 | 1.25 | 0.38 |  | 0 | 0.38 | 1.89 | 0 |  | 0 | 0.25 | 1.75 | 0.25 |  | 0 | 0 | 0 | 0 |  | 0 | 0 | 0 | 0 |  | 0 | 0 | 1 | 0.4 |  | 0 | 0.5 | 0 | 0 | 0 | 2 | 4 | 0 |
| 22 | 0.04 1.17 | 1.63 | 0.75 |  | 0.11 | 0.67 | 1.89 | 0 |  | 0 | 0.25 | 1.75 | 1.25 |  | 0 | 0 | 0 | 1 |  | 0 | 0 | 1 | 0 |  | 0 | 1 | 1 | 1.2 |  | 0.25 | 0.5 | 0.25 | 0.25 | 0 | 5 | 26 | 1 |
| X | 0 0.63 | 2.08 | 0.29 |  | 0 | 0.75 | 1.56 | 0 |  | 0 | 0.25 | 6.5 | 0.5 |  | 0 | 0 | 0 | 0 |  | 0 | 0 | 0.5 | 0 |  | 0 | 0.5 | 1.4 | 1 |  | 0 | 0 | 0 | 0 | 0 | 0 | 0 | 0 |
| HD, homozygous deletion; HL, heterozygous loss; LLG, low-level gain; HLA, high-level amplification | | | | | | | | | | | | | | | | | |  | | |  | |  | | |  | |  | | |  | |  |  |  | | |
