# Supplementary material for: Genetic alterations associated with multiple primary malignancies
Source: Cancer Med. 2021 May 31;10(13):4465–77. doi: 10.1002/cam4.3975 (PMC8267160; doi:10.1002/cam4.3975)
Supplement: Supplementary file 2 — Table S2 [file CAM4-10-4465-s002.docx]

| **Supplementary Table 2. Recurrent regions of LOH in the tumor samples** | | | | | | | | | | | | | | | | | | | | |
| --- | --- | --- | --- | --- | --- | --- | --- | --- | --- | --- | --- | --- | --- | --- | --- | --- | --- | --- | --- | --- |
|  |  |  |  |  |  |  |  |  |  |  |  |  |  |  |  |  |  |  |  |  |
| **Chrom** | **BC (n=24)** | | | | **MM (n=9)** | | | | **HM (n=4)** | | | | **GYM (n=4)** | | | | **OM (n=2)** | | | |
|  | Mean | Max | Min | SEM | Mean | Max | Min | SEM | Mean | Max | Min | SEM | Mean | Max | Min | SEM | Mean | Max | Min | SEM |
| Total | 23501 | 102646 | 1551 | 7550.5 | 26644 | 100724 | 3039 | 48842.5 | 4326.25 | 7237 | 2548 | 2344.5 | 21638.75 | 63982 | 3698 | 30142 | 24683.5 | 30612 | 18755 | 5928.5 |
| 1 | 3662 | 15205 | 104 | 4070 | 2806 | 7429 | 0 | 3714.5 | 1143 | 2067 | 0 | 1033.5 | 833 | 2002 | 229 | 886.5 | 1726 | 3212 | 240 | 1486 |
| 2 | 1881 | 8274 | 134 | 4070 | 2770 | 9469 | 0 | 4734.5 | 337 | 620 | 0 | 310 | 6157 | 17087 | 0 | 8543.5 | 1613 | 3058 | 168 | 1445 |
| 3 | 1523 | 7084 | 177 | 3453.5 | 2516 | 8068 | 0 | 4034 | 252 | 252 | 0 | 126 | 3871 | 7412 | 0 | 3706 | 0 | 0 | 0 | 0 |
| 4 | 3298 | 7655 | 169 | 3743 | 3124 | 9354 | 0 | 4677 | 0 | 0 | 0 | 0 | 202 | 202 | 0 | 101 | 0 | 0 | 0 | 0 |
| 5 | 2775 | 9100 | 197 | 4451.5 | 1457 | 5699 | 196 | 2751.5 | 188 | 188 | 0 | 94 | 3163 | 9119 | 0 | 4559.5 | 1288 | 2105 | 470 | 817.5 |
| 6 | 3203 | 12475 | 184 | 6145.5 | 2522 | 6205 | 0 | 3102.5 | 1373 | 3480 | 0 | 1740 | 588 | 783 | 0 | 391.5 | 3760 | 7003 | 517 | 3243 |
| 7 | 795 | 2357 | 85 | 1136 | 1297 | 6072 | 0 | 3036 | 770 | 770 | 0 | 385 | 351 | 504 | 0 | 252 | 3446 | 6315 | 576 | 2869.5 |
| 8 | 2432 | 10736 | 169 | 5283.5 | 1795 | 5837 | 0 | 2918.5 | 259 | 259 | 0 | 129.5 | 410 | 701 | 0 | 350.5 | 266 | 266 | 0 | 133 |
| 9 | 3083 | 6151 | 504 | 2823.5 | 3523 | 4013 | 0 | 2006.5 | 0 | 0 | 0 | 0 | 3062 | 3062 | 0 | 1531 | 704 | 704 | 0 | 352 |
| 10 | 1769 | 4750 | 236 | 2257 | 1273 | 4016 | 0 | 2008 | 0 | 0 | 0 | 0 | 248 | 294 | 0 | 147 | 427.5 | 614 | 241 | 186.5 |
| 11 | 2840 | 7710 | 311 | 3699.5 | 3411 | 9944 | 0 | 4972 | 399 | 506 | 0 | 253 | 1681 | 3029 | 0 | 1514.5 | 3851 | 3851 | 0 | 1925.5 |
| 12 | 2018 | 6498 | 175 | 3161.5 | 1797 | 4756 | 0 | 2378 | 220 | 254 | 0 | 127 | 771 | 1819 | 0 | 909.5 | 2685 | 4428 | 941 | 1743.5 |
| 13 | 3702 | 7931 | 404 | 3763.5 | 2748 | 6706 | 0 | 3353 | 376 | 376 | 0 | 188 | 362 | 362 | 0 | 181 | 542 | 542 | 0 | 271 |
| 14 | 1764 | 4119 | 183 | 1968 | 1515 | 2601 | 0 | 1300.5 | 292 | 384 | 0 | 192 | 0 | 0 | 0 | 0 | 0 | 0 | 0 | 0 |
| 15 | 2028 | 6003 | 164 | 2919.5 | 1730 | 4104 | 0 | 2052 | 216 | 246 | 0 | 123 | 196 | 196 | 0 | 98 | 0 | 0 | 0 | 0 |
| 16 | 1367 | 3774 | 111 | 1831.5 | 1065 | 3760 | 111 | 1824.5 | 216 | 398 | 0 | 199 | 198 | 274 | 0 | 137 | 1751 | 3154 | 347 | 1403.5 |
| 17 | 2501 | 6085 | 122 | 2981.5 | 1245 | 3329 | 0 | 1664.5 | 578 | 578 | 0 | 289 | 1348 | 2027 | 0 | 1013.5 | 836 | 1290 | 382 | 454 |
| 18 | 2684 | 5594 | 1072 | 2261 | 1199 | 2142 | 0 | 1071 | 0 | 0 | 0 | 0 | 0 | 0 | 0 | 0 | 0 | 0 | 0 | 0 |
| 19 | 1016 | 2006 | 251 | 877.5 | 1000 | 2723 | 0 | 1361.5 | 0 | 0 | 0 | 0 | 0 | 0 | 0 | 0 | 902 | 1553 | 251 | 651 |
| 20 | 3412 | 5115 | 257 | 2429 | 1718 | 4282 | 0 | 2141 | 0 | 0 | 0 | 0 | 5115 | 5115 | 0 | 2557.5 | 1417 | 1417 | 0 | 708.5 |
| 21 | 740 | 1795 | 166 | 814.5 | 213 | 213 | 0 | 106.5 | 0 | 0 | 0 | 0 | 0 | 0 | 0 | 0 | 0 | 0 | 0 | 0 |
| 22 | 1689 | 2794 | 280 | 1257 | 958 | 1681 | 0 | 840.5 | 0 | 0 | 0 | 0 | 670 | 670 | 0 | 335 | 579 | 579 | 0 | 289.5 |
| X | 1993 | 10189 | 517 | 4836 | 3067 | 11207 | 0 | 5603.5 | 1021.5 | 1653 | 0 | 826.5 | 3606.5 | 11207 | 0 | 5603.5 | 2563 | 4209 | 916 | 1646.5 |
|  |  |  |  |  |  |  |  |  |  |  |  |  |  |  |  |  |  |  |  |  |
|  |  |  |  |  |  |  |  |  |  |  |  |  |  |  |  |  |  |  |  |  |
| **Chrom** | **TM (n=2)** | | | | **Sarcoma (n=1)** | | | | **GIM (n=1)** | | | |  |  |  |  |  |  |  |  |
|  | Mean | Max | Min | SEM | Mean | Max | Min | SEM | Mean | Max | Min | SEM |  |  |  |  |  |  |  |  |
| Total | 3364 | 3631 | 3097 | 267 | 5997 | 5997 | 5997 | 0 | 6081 | 6081 | 6081 | 0 |  |  |  |  |  |  |  |  |
| 1 | 96 | 96 | 0 | 48 | 194 | 194 | 194 | 0 | 172 | 172 | 172 | 0 |  |  |  |  |  |  |  |  |
| 2 | 211 | 211 | 0 | 105.5 | 0 | 0 | 0 | 0 | 257 | 257 | 257 | 0 |  |  |  |  |  |  |  |  |
| 3 | 358 | 395 | 321 | 37 | 0 | 0 | 0 | 0 | 0 | 0 | 0 | 0 |  |  |  |  |  |  |  |  |
| 4 | 0 | 0 | 0 | 0 | 0 | 0 | 0 | 0 | 182 | 182 | 182 | 0 |  |  |  |  |  |  |  |  |
| 5 | 0 | 0 | 0 | 0 | 0 | 0 | 0 | 0 | 0 | 0 | 0 | 0 |  |  |  |  |  |  |  |  |
| 6 | 174 | 174 | 0 | 87 | 2187 | 2187 | 2187 | 0 | 224 | 224 | 224 | 0 |  |  |  |  |  |  |  |  |
| 7 | 172 | 172 | 0 | 86 | 279 | 279 | 279 | 0 | 88 | 88 | 88 | 0 |  |  |  |  |  |  |  |  |
| 8 | 249 | 249 | 0 | 124.5 | 202 | 202 | 202 | 0 | 549 | 549 | 549 | 0 |  |  |  |  |  |  |  |  |
| 9 | 0 | 0 | 0 | 0 | 0 | 0 | 0 | 0 | 178 | 178 | 178 | 0 |  |  |  |  |  |  |  |  |
| 10 | 0 | 0 | 0 | 0 | 0 | 0 | 0 | 0 | 1394 | 1394 | 1394 | 0 |  |  |  |  |  |  |  |  |
| 11 | 244 | 290 | 198 | 46 | 844 | 844 | 844 | 0 | 714 | 714 | 714 | 0 |  |  |  |  |  |  |  |  |
| 12 | 441 | 441 | 0 | 220.5 | 208 | 208 | 208 | 0 | 0 | 0 | 0 | 0 |  |  |  |  |  |  |  |  |
| 13 | 0 | 0 | 0 | 0 | 0 | 0 | 0 | 0 | 0 | 0 | 0 | 0 |  |  |  |  |  |  |  |  |
| 14 | 183 | 183 | 0 | 91.5 | 0 | 0 | 0 | 0 | 0 | 0 | 0 | 0 |  |  |  |  |  |  |  |  |
| 15 | 0 | 0 | 0 | 0 | 170 | 170 | 170 | 0 | 0 | 0 | 0 | 0 |  |  |  |  |  |  |  |  |
| 16 | 102 | 113 | 91 | 11 | 331 | 331 | 331 | 0 | 111 | 111 | 111 | 0 |  |  |  |  |  |  |  |  |
| 17 | 0 | 0 | 0 | 0 | 435 | 435 | 435 | 0 | 0 | 0 | 0 | 0 |  |  |  |  |  |  |  |  |
| 18 | 0 | 0 | 0 | 0 | 0 | 0 | 0 | 0 | 0 | 0 | 0 | 0 |  |  |  |  |  |  |  |  |
| 19 | 0 | 0 | 0 | 0 | 0 | 0 | 0 | 0 | 0 | 0 | 0 | 0 |  |  |  |  |  |  |  |  |
| 20 | 0 | 0 | 0 | 0 | 0 | 0 | 0 | 0 | 190 | 190 | 190 | 0 |  |  |  |  |  |  |  |  |
| 21 | 0 | 0 | 0 | 0 | 0 | 0 | 0 | 0 | 0 | 0 | 0 | 0 |  |  |  |  |  |  |  |  |
| 22 | 249.5 | 287 | 212 | 37.5 | 0 | 0 | 0 | 0 | 0 | 0 | 0 | 0 |  |  |  |  |  |  |  |  |
| X | 1647.5 | 1754 | 1541 | 106.5 | 1147 | 1147 | 1147 | 0 | 2022 | 2022 | 2022 | 0 |  |  |  |  |  |  |  |  |
