# Supplementary material for: Genetic alterations associated with multiple primary malignancies
Source: Cancer Med. 2021 May 31;10(13):4465–77. doi: 10.1002/cam4.3975 (PMC8267160; doi:10.1002/cam4.3975)
Supplement: Supplementary file 3 — Table S3 [file CAM4-10-4465-s004.docx]

| **Supplementary Table 3. Reported clinical significance of SNPs associated with the identified mutations** | | | | | | | | |
| --- | --- | --- | --- | --- | --- | --- | --- | --- |
|  |  |  |  |  |  |  |  |  |
| **Mutation** | **Patient** | **Tumor** | **Chromosome** | **Reported clinical significance according to dbSNP^†^** | | | | |
|  |  |  |  | *Disease name* | *SNP* | *Clinical significance* | *Somatic/ Germline/ SNV* | *Consequence* |
| BRAF:p.V600E:c.1799T>A | 13A | Breast | 7 | Papillary thyroid carcinoma | rs113488022 | Pathogenic | Somatic | Missense |
|  | 19B | Malignant Melanoma |  | Cutaneous melanoma |  | Pathogenic |  |  |
|  |  |  |  | Cardio-facio-cutaneous syndrome |  | Pathogenic |  |  |
|  |  |  |  | Carcinoma of colon |  | Pathogenic |  |  |
|  |  |  |  | Gastrointestinal stroma tumor |  | Pathogenic |  |  |
|  |  |  |  | Lung adenocarcinoma |  | Pathogenic |  |  |
|  |  |  |  | Non-small cell lung cancer |  | Pathogenic |  |  |
|  |  |  |  | Ovarian Neoplasms |  | Pathogenic |  |  |
|  |  |  |  | Germ cell tumor. nonseminomatous |  | Pathogenic |  |  |
|  |  |  |  | Astrocytoma. low-grade. somatic |  | Pathogenic |  |  |
|  |  |  |  | Brainstem glioma |  | Likely pathogenic |  |  |
|  |  |  |  | Glioblastoma |  | Likely pathogenic |  |  |
|  |  |  |  | Neoplasm of brain |  | Likely pathogenic |  |  |
|  |  |  |  | Papillary renal cell carcinoma. sporadic |  | Likely pathogenic |  |  |
|  |  |  |  | Multiple myeloma |  | Likely pathogenic |  |  |
|  |  |  |  | Squamous cell carcinoma of the head and neck |  | Likely pathogenic |  |  |
| IDH2:p.R140Q:c.419G>A | 07A | Breast | 15 | Myelodysplastic syndrome | rs121913502 | Likely pathogenic | SNV | Missense |
|  |  |  |  | Acute myeloid leukemia |  | Pathogenic |  |  |
|  |  |  |  | Squamous cell carcinoma of the head and neck |  | Likely pathogenic |  |  |
|  |  |  |  | Neoplasm of the large intestine |  | Likely pathogenic |  |  |
|  |  |  |  | Multiple myeloma |  | Likely pathogenic |  |  |
| KRAS:p.G13D:c.38G>A | 06B | Adenocarcinom Ceacum | 12 | Breast adenocarcinoma | rs121913529 | Pathogenic | Somatic | Missense |
|  |  |  |  | Non-small cell lung cancer |  | Pathogenic |  |  |
|  |  |  |  | Neoplasm of the thyroid gland |  | Likely pathogenic |  |  |
|  |  |  |  | Neoplasm of the large intestine |  | Pathogenic |  |  |
|  |  |  |  | Juvenile myelomonocytic leukemia |  | Pathogenic |  |  |
|  |  |  |  | RAS-associated autoimmune leukoproliferative disorder |  | Pathogenic |  |  |
|  |  |  |  | Acute myeloid leukemia |  | Likely pathogenic |  |  |
|  |  |  |  | Ovarian Neoplasms |  | Pathogenic |  |  |
| NRAS:p.Q61R:c.182A>G | 04B | Thyroid carcinoma | 1 | Malignant neoplasm of body of uterus | rs11554290 | Likely pathogenic | SNV | Missense |
|  |  |  |  | Ovarian Serous Cystadenocarcinoma |  | Likely pathogenic |  |  |
|  |  |  |  | Adenocarcinoma of stomach |  | Likely pathogenic |  |  |
|  |  |  |  | Hepatocellular carcinoma |  | Likely pathogenic |  |  |
|  |  |  |  | Neoplasm of the large intestine |  | Likely pathogenic |  |  |
|  |  |  |  | Glioblastoma |  | Likely pathogenic |  |  |
|  |  |  |  | Neoplasm of brain |  | Likely pathogenic |  |  |
|  |  |  |  | Acute myeloid leukemia |  | Likely pathogenic |  |  |
|  |  |  |  | Multiple myeloma |  | Likely pathogenic |  |  |
|  |  |  |  | Chronic lymphocytic leukemia |  | Likely pathogenic |  |  |
|  |  |  |  | Renal cell carcinoma. Papillary |  | Likely pathogenic |  |  |
|  |  |  |  | Adrenocortical carcinoma |  | Likely pathogenic |  |  |
|  |  |  |  | Neoplasm of the thyroid gland |  | Likely pathogenic |  |  |
|  |  |  |  | Nasopharyngeal Neoplasms |  | Likely pathogenic |  |  |
|  |  |  |  | Lung adenocarcinoma |  | Likely pathogenic |  |  |
|  |  |  |  | Non-small cell lung cancer |  | Pathogenic |  |  |
|  |  |  |  | Malignant melanoma |  | Pathogenic |  |  |
|  |  |  |  | Transitional cell carcinoma of the bladder |  | Likely pathogenic |  |  |
| PIK3CA:p.H1047R:c.3140A>G | 15A | Breast | 3 | Breast adenocarcinoma | rs121913279 | Pathogenic | Somatic | Missense |
|  |  |  |  | Ovarian epithelial cancer |  | Pathogenic |  |  |
|  |  |  |  | Ovarian Serous Cystadenocarcinoma |  | Likely pathogenic |  |  |
|  |  |  |  | Uterine Carcinosarcoma |  | Likely pathogenic |  |  |
|  |  |  |  | Carcinoma of colon |  | Pathogenic |  |  |
|  |  |  |  | Carcinoma of esophagus |  | Likely pathogenic |  |  |
|  |  |  |  | Adenocarcinoma of stomach |  | Pathogenic |  |  |
|  |  |  |  | Hepatocellular carcinoma |  | Pathogenic |  |  |
|  |  |  |  | Non-small cell lung cancer |  | Pathogenic |  |  |
|  |  |  |  | Neoplasm of ovary |  | Pathogenic |  |  |
|  |  |  |  | Malignant melanoma of skin |  | Likely pathogenic |  |  |
|  |  |  |  | Pancreatic adenocarcinoma |  | Likely pathogenic |  |  |
|  |  |  |  | Medulloblastoma |  | Likely pathogenic |  |  |
|  |  |  |  | Lung adenocarcinoma |  | Likely pathogenic |  |  |
|  |  |  |  | Squamous cell lung carcinoma |  | Likely pathogenic |  |  |
|  |  |  |  | Brainstem glioma. Neoplasm of brain |  | Likely pathogenic |  |  |
|  |  |  |  | Adrenocortical carcinoma |  | Likely pathogenic |  |  |
|  |  |  |  | Squamous cell carcinoma of the head and neck |  | Likely pathogenic |  |  |
|  |  |  |  | Adenocarcinoma of prostate |  | Likely pathogenic |  |  |
| TP53:p.G245S/C:c.733G>A/T | 3B | Malignant Melanoma in Situ | 17 | Li-Fraumeni syndrome 1 | rs28934575 | Pathogenic | Germline/ Somatic/ Sporadic | Missense |
|  | 6A | Breast |  | Uterine Carcinosarcoma |  | Likely pathogenic |  |  |
|  | 7A | Breast |  | Lung adenocarcinoma |  | Likely pathogenic |  |  |
|  | 8A | Breast |  | Neoplasm of the breast |  | Likely pathogenic |  |  |
|  | 10B | Mb Hodgins |  | Ovarian Serous Cystadenocarcinoma |  | Likely pathogenic |  |  |
|  | 11A | Breast |  | Adenocarcinoma of stomach |  | Likely pathogenic |  |  |
|  | 12B | Tounge squamous cell carcinoma |  | Glioblastoma |  | Likely pathogenic |  |  |
|  |  |  |  | Transitional cell carcinoma of the bladder |  | Likely pathogenic |  |  |
|  |  |  |  | Carcinoma of esophagus |  | Likely pathogenic |  |  |
|  |  |  |  | Hepatocellular carcinoma |  | Likely pathogenic |  |  |
|  |  |  |  | Neoplasm of the large intestine |  | Likely pathogenic |  |  |
|  |  |  |  | Pancreatic adenocarcinoma |  | Likely pathogenic |  |  |
|  |  |  |  | Adenocarcinoma of prostate |  | Likely pathogenic |  |  |
|  |  |  |  | Brainstem glioma |  | Likely pathogenic |  |  |
|  |  |  |  | Squamous cell carcinoma of the head and neck |  | Likely pathogenic |  |  |
|  |  |  |  | Squamous cell lung carcinoma |  | Likely pathogenic |  |  |
|  |  |  |  | Neoplasm of brain |  | Likely pathogenic |  |  |
|  |  |  |  | Astrocytoma. anaplastic |  | Pathogenic |  |  |
|  |  |  |  | Hereditary cancer-predisposing syndrome |  | Pathogenic |  |  |
| TP53:p.R175H:c.524G>A | 5A | Breast | 17 | Li-Fraumeni syndrome | rs28934578 | Pathogenic | Germline/ Somatic/ Sporadic | Missense |
|  | 17B | Uterus leomyosarcoma |  | Hereditary cancer-predisposing syndrome |  | Pathogenic |  |  |
|  | 21A | Breast |  | Malignant tumor of esophagus |  | Pathogenic |  |  |
|  |  |  |  | Neoplasm of the breast |  | Pathogenic |  |  |
|  |  |  |  | Adrenocortical carcinoma |  | Pathogenic |  |  |
|  |  |  |  | Basal cell carcinoma |  | Pathogenic |  |  |
|  |  |  |  | Carcinoma of colon |  | Pathogenic |  |  |
|  |  |  |  | Familial cancer of breast |  | Pathogenic |  |  |
|  |  |  |  | Osteosarcoma |  | Pathogenic |  |  |
|  |  |  |  | Nasopharyngeal carcinoma |  | Pathogenic |  |  |
|  |  |  |  | Ovarian Neoplasms |  | Likely pathogenic |  |  |
| TP53:p.Y163C:c.488A>G | 21A | Breast | 17 | Neoplasm of the large intestine | rs148924904 | Likely pathogenic | Germline/ Somatic/ Sporadic | Missense |
|  |  |  |  | Hepatocellular carcinoma |  | Likely pathogenic |  |  |
|  |  |  |  | Pancreatic adenocarcinoma |  | Likely pathogenic |  |  |
|  |  |  |  | Carcinoma of esophagus |  | Likely pathogenic |  |  |
|  |  |  |  | Squamous cell lung carcinoma |  | Likely pathogenic |  |  |
|  |  |  |  | Lung adenocarcinoma |  | Likely pathogenic |  |  |
|  |  |  |  | Small cell lung cancer |  | Likely pathogenic |  |  |
|  |  |  |  | Uterine Carcinosarcoma |  | Likely pathogenic |  |  |
|  |  |  |  | Ovarian Serous Cystadenocarcinoma |  | Likely pathogenic |  |  |
|  |  |  |  | Ovarian Neoplasms |  | Likely pathogenic |  |  |
|  |  |  |  | Neoplasm of the breast |  | Likely pathogenic |  |  |
|  |  |  |  | Malignant melanoma of skin |  | Likely pathogenic |  |  |
|  |  |  |  | Brainstem glioma |  | Likely pathogenic |  |  |
|  |  |  |  | Neoplasm of brain |  | Likely pathogenic |  |  |
|  |  |  |  | Squamous cell carcinoma of the head and neck |  | Likely pathogenic |  |  |
|  |  |  |  | Hereditary cancer-predisposing syndrome |  | Pathogenic |  |  |
|  |  |  |  | Li-Fraumeni syndrome |  | Pathogenic |  |  |
| TP53:p.Y220C:c.659A>G | 21A | Breast | 17 | Hereditary cancer-predisposing syndrome | rs121912666 | Pathogenic | Germline/ Somatic/ Sporadic | Missense |
|  |  |  |  |  |  |  |  |  |
|  | 21B | Malignant Melanoma |  | Li-Fraumeni syndrome |  | Pathogenic |  |  |
|  | 23B | Mb Hodgins |  | Glioblastoma |  | Likely pathogenic |  |  |
|  |  |  |  | Ovarian Serous Cystadenocarcinoma |  | Likely pathogenic |  |  |
|  |  |  |  | Malignant neoplasm of body of uterus |  | Likely pathogenic |  |  |
|  |  |  |  | Uterine Carcinosarcoma |  | Likely pathogenic |  |  |
|  |  |  |  | Ovarian Neoplasms |  | Likely pathogenic |  |  |
|  |  |  |  | Neoplasm of the breast |  | Likely pathogenic |  |  |
|  |  |  |  | Squamous cell carcinoma of the head and neck |  | Likely pathogenic |  |  |
|  |  |  |  | Malignant melanoma of skin |  | Likely pathogenic |  |  |
|  |  |  |  | Transitional cell carcinoma of the bladder |  | Likely pathogenic |  |  |
|  |  |  |  | Papillary renal cell carcinoma. sporadic |  | Likely pathogenic |  |  |
|  |  |  |  | Renal cell carcinoma. Papillary |  | Likely pathogenic |  |  |
|  |  |  |  | Adenocarcinoma of prostate |  | Likely pathogenic |  |  |
|  |  |  |  | Neoplasm of brain |  | Likely pathogenic |  |  |
|  |  |  |  | Acute myeloid leukemia |  | Likely pathogenic |  |  |
|  |  |  |  | Lung adenocarcinoma |  | Likely pathogenic |  |  |
|  |  |  |  | Small cell lung cancer |  | Likely pathogenic |  |  |
|  |  |  |  | Squamous cell lung carcinoma |  | Likely pathogenic |  |  |
|  |  |  |  | Adenocarcinoma of stomach |  | Likely pathogenic |  |  |
|  |  |  |  | Hepatocellular carcinoma |  | Likely pathogenic |  |  |
|  |  |  |  | Pancreatic adenocarcinoma |  | Likely pathogenic |  |  |
|  |  |  |  | Neoplasm of the large intestine |  | Likely pathogenic |  |  |
| ^†^https://www.ncbi.nlm.nih.gov/snp | |  |  |  |  |  |  |  |
