# Supplementary material for: Genetic alterations associated with multiple primary malignancies
Source: Cancer Med. 2021 May 31;10(13):4465–77. doi: 10.1002/cam4.3975 (PMC8267160; doi:10.1002/cam4.3975)
Supplement: Supplementary file 4 — Table S4 [file CAM4-10-4465-s001.docx]

Supplementary Table 4. Genetic instability according to GII, CAII, and CTLP

| Patient | Sample | Type of cancer | GII  Stable: 0 (<0.2)  Unstable: 1 (>0.2) | Complex armwise abberation (CAAI) (stable <0.5, unstable >0.5) | | | Chromotripsis p≤0.05,>20 times |
| --- | --- | --- | --- | --- | --- | --- | --- |
|  |  |  |  | *Stable/Unstable* | *mean* | *max* |  |
| 1 | A | Breast | 0 | Unstable | 1.39 | 4.55 | 0 |
|  | *B* | *MM in situ* | *no sample* | | | | |
| *2 (excluded)* | *X* | *Breast* |  |  |  |  |  |
|  | *X* | *MM in situ* |  |  |  |  |  |
| 3 | A | Breast | 0 | Unstable | 0.05 | 0.84 | 0 |
|  | B | MM in situ | 0 | Unstable | 0.02 | 0.73 | 0 |
| 4 | A | Breast | 0 | Unstable | 0.36 | 5.29 | 0 |
|  | B | Thyroid ca | 0 | Unstable | 0.079 | 2.72 | 0 |
| 5 | A | Breast | 1 | Unstable | 0.45 | 2.62 | 0 |
|  | B | MM in situ | 0 | Unstable | 0.06 | 1.35 | 0 |
| 6 | A | Breast | 1 | Unstable | 0.01 | 0.34 | 0 |
|  | B | Adenocarcinom ceacum | 0 | Unstable | 0.02 | 0.64 | 0 |
| 7 | A | Breast | 0 | Unstable | 0.53 | 6.3 | event on chr 17 |
|  |  |  | 0 | Unstable |  |  | event on chr 17 |
|  | B | MM | 0 | Unstable | 0.02 | 0.80 | 0 |
| 8 | A | Breast | 0 | Unstable | 0.81 | 20.20 | event on chr 10 & 11 |
|  | B | Ovarial mucinous cystadenocarcinom | 1 | Unstable | 0.14 | 1.35 | 0 |
| 9 | A | Breast | 0 | Unstable | 0.04 | 1.05 | 0 |
|  | B | MM in situ | 1 | Unstable | 0.03 | 1.30 | 0 |
| 10 | A | Breast | 1 | Unstable | 0.37 | 12.57 | event on chr 17 |
|  | B | Mb Hodgins | 0 | Unstable | 0.09 | 2.37 | 0 |
| 11 | A | Breast | 0 | Unstable | 0.19 | 3.72 | 0 |
|  | B | Sarcoma | 0 | Unstable | 1.06 | 6.96 | event on chr 19 |
| 12 | A | Breast | 0 | Unstable | 0.10 | 1.47 | 0 |
|  | B | Tounge squamous cell carcinoma | 1 | Unstable | 0 | 0.01 | 0 |
| 13 | A | Breast | 0 | Unstable | 0.01 | 0.54 | 0 |
|  | B | thyroid | 0 | Unstable | 0.02 | 0.68 | 0 |
| 14 | A | Breast | 0 | Unstable | 0.02 | 0.92 | 0 |
|  | B | MM in situ | 0 | Unstable | 0.03 | 0.89 | 0 |
| 15 | A | Breast | 0 | Unstable | 0.07 | 1.35 | 0 |
|  | B | MM | 0 | Unstable | 0 | 0.01 | 0 |
| *16*  *(excluded)* | *A* | *Breast* |  |  |  |  |  |
|  | *B* | *MM in situ* |  |  |  |  |  |
| 17 | A | Breast | 0 | Unstable | 0.05 | 1.98 | 0 |
|  | B | Uterine leiomyosarcoma | 0 | Unstable | 0.057 | 1.50 | 0 |
| 18 | A | Breast | 0 | Unstable | 0.05 | 1.05 | 0 |
|  | B | Oral Cavity | 0 | Unstable | 0.01 | 0.26 | 0 |
| 19 | A | Breast | 0 | Unstable | 1.94 | 8.77 | 0 |
|  | B | MM | 1 | Unstable | 3.47 | 9.63 | 0 |
| 20 | A | Breast | 0 | Unstable | 0.07 | 1.09 | 0 |
|  | B | Lymphoma | 0 | Unstable | 0.05 | 1.60 | 0 |
| 21 | A | Breast | 1 | Unstable | 0.75 | 5.25 | 0 |
|  |  |  | 1 | Unstable |  |  | 0 |
|  |  |  | 1 | Unstable |  |  | 0 |
|  | B | MM | 1 | Unstable | 0.13 | 2.52 |  |
| 22 | A | Breast | 0 | Unstable | 0.15 | 2.08 | 0 |
|  | B | MM | 1 | Unstable | 0.07 | 2.19 | 0 |
| 23 | A | Breast | 0 | Unstable | 0.22 | 1.80 | 0 |
|  | B | Hodgins lymphoma | 0 | Unstable | 0.24 | 2.69 | 0 |
| 24 | A | Breast | 0 | Unstable | 0.47 | 11.31 | event chr 4 & 10 |
|  | B | Lymphoma | 0 | Unstable | 1.83 | 9.48 | 0 |
| 25 | A | Breast | 0 | Unstable | 0.24 | 2.23 | 0 |
|  | B | Cervix | 0 | Unstable | 0.07 | 3.43 | 0 |
|  | *C* | *Excluded* |  |  |  |  |  |
| 26 | A | Breast | 0 | Unstable | 4.05 | 9.18 | 0 |
|  | B | Adenocarcinoma in situ cervix | 1 | Unstable | 8.22 | 16.03 | 0 |
